# Variants at 8p21.3 Mediate *BMP1* Expression, Contributing to Higher Atopic Dermatitis Risk

**DOI:** 10.1111/cea.70332

**Published:** 2026-05-08

**Authors:** Jia Yi Karen Wong, Yang Yie Sio, Yi Ying Eliza Lim, Yee‐How Say, Kavita Reginald, Fook Tim Chew

**Affiliations:** ^1^ Department of Biological Sciences, Faculty of Science National University of Singapore Singapore Singapore; ^2^ Department of Biomedical Sciences, Sir Jeffrey Cheah Sunway Medical School, Faculty of Medical and Life Sciences Sunway University Petaling Jaya Malaysia; ^3^ Department of Biomedical Science, Faculty of Science Universiti Tunku Abdul Rahman (UTAR) Kampar Perak Malaysia

## Abstract

- 8p21.3 polymorphisms associate with lower *BMP1* expression, lower skin hydration and increased AD susceptibility.
- Regulatory variants at 8p21.3 reduce *BMP1* promoter activity in dermal fibroblasts, mediating its downregulation.

To the Editor,

Atopic dermatitis (AD) is a chronic inflammatory skin disorder in which epidermal barrier dysfunction and immune dysregulation jointly drive disease susceptibility and progression [1]. While these mechanisms have been extensively studied, increasing literature highlights the roles of extracellular matrix (ECM) components, particularly collagens, in AD [2]. AD patients have been reported to exhibit lower type I and type III collagen expression in the skin compared to healthy individuals [3], while lesions featured reduced type IV collagen and a thinner basement membrane [4]. These studies suggest that disruptions in collagen composition may compromise barrier integrity, facilitating allergen/irritant penetration [3, 5]. Bone morphogenetic protein 1 (BMP1) is a secreted metalloprotease known for its pivotal role in ECM assembly, where it cleaves carboxyl‐terminal propeptides of procollagens to produce mature collagen fibrils, thereby maintaining the structural integrity of connective tissues [6]. In murine models, *BMP1* deficiency led to thinned and fragile skin with reduced mechanical strength [6]. Despite its central role in skin biology, BMP1 has received little attention in the context of chronic skin diseases. This study evaluates the potential involvement of BMP1 in AD pathogenesis by (i) profiling *BMP1* expression in peripheral blood mononuclear cells (PBMCs) from participants of the Singapore/Malaysia Cross‐Sectional Genetic Epidemiology Study (SMCGES), (ii) identifying proximal cis‐regulatory variants at 8p21.3 affecting *BMP1* expression, (iii) functionally validating promoter activity and (iv) assessing the relationship between *BMP1* and skin barrier function.

We analysed participants of Chinese ethnicity from the SMCGES. Details of participant recruitment have been reported previously [7]. Two independent cohorts were used (Repository Table S1): a transcriptome‐profiled cohort in which PBMCs were collected for whole‐transcriptome sequencing and single nucleotide polymorphism (SNP) genotyping (*n* = 518; Age = 22.8 ± 7.2; 52.3% females), and a genotyped cohort that provided buccal cells for SNP genotyping (*n* = 2742; Age = 22.7 ± 6.2; 65.0% females). AD status was determined using validated diagnostic guidelines (Hanifin and Rajka, and the UK Working Party criteria) and sensitisation to common house dust mites assessed by skin prick testing (SPT). Individuals were classified as having AD if they reported an itchy rash lasting or recurring for at least 6 months affecting flexural areas, and had a positive SPT response (wheal diameter ≥ 3 mm) to *Dermatophagoides pteronyssinus* or *Blomia tropicalis*. Participants who reported no symptoms of AD and exhibited a negative SPT response were classified as non‐AD controls. AD cases were further stratified by severity based on sleep disturbance in the past 12 months—mild (never kept awake), moderate (less than one night per week) and severe (one or more nights per week).

In PBMCs, *BMP1* transcript expression was significantly lower in AD individuals compared with non‐AD controls (*p* < 0.01) and declined stepwise with increasing disease severity, with the highest levels in non‐AD controls, followed by mild AD (*p* < 0.05 versus controls), and then moderate AD (*p* < 0.01 versus mild AD; Figure 1A). To evaluate whether proximal cis‐regulatory variation contributes to lower *BMP1* expression, we examined common variants within ± 2 kb of *BMP1* (chr8: 22,020,885–22,071,839; GRCh37/hg19) using the 1000 Genomes Han Chinese in Beijing, China (CHB) reference panel (Repository Figure S1A). In this region, 108 SNPs with minor allele frequencies (MAFs) ≥ 5% were captured by 35 tag SNPs (LD *r* ^*2*^ ≥ 0.8; Repository Table S2), of which 33 were successfully genotyped and tested for association with *BMP1* levels in PBMCs (Repository Table S3). Among the three tag SNPs (rs4715, rs4348488 and rs7838961), only rs4348488 exhibited a significant association with AD risk (Repository Figure S1B). Its minor allele ‘C’ conferred a 1.20‐fold higher odds of AD (95% CI: 1.07–1.34; *p* < 0.01) after adjustments for age and gender, and was significantly associated with lower *BMP1* expression (*p* < 0.001). These findings support a model in which lower *BMP1* expression may contribute to increased AD susceptibility.

**FIGURE 1:**
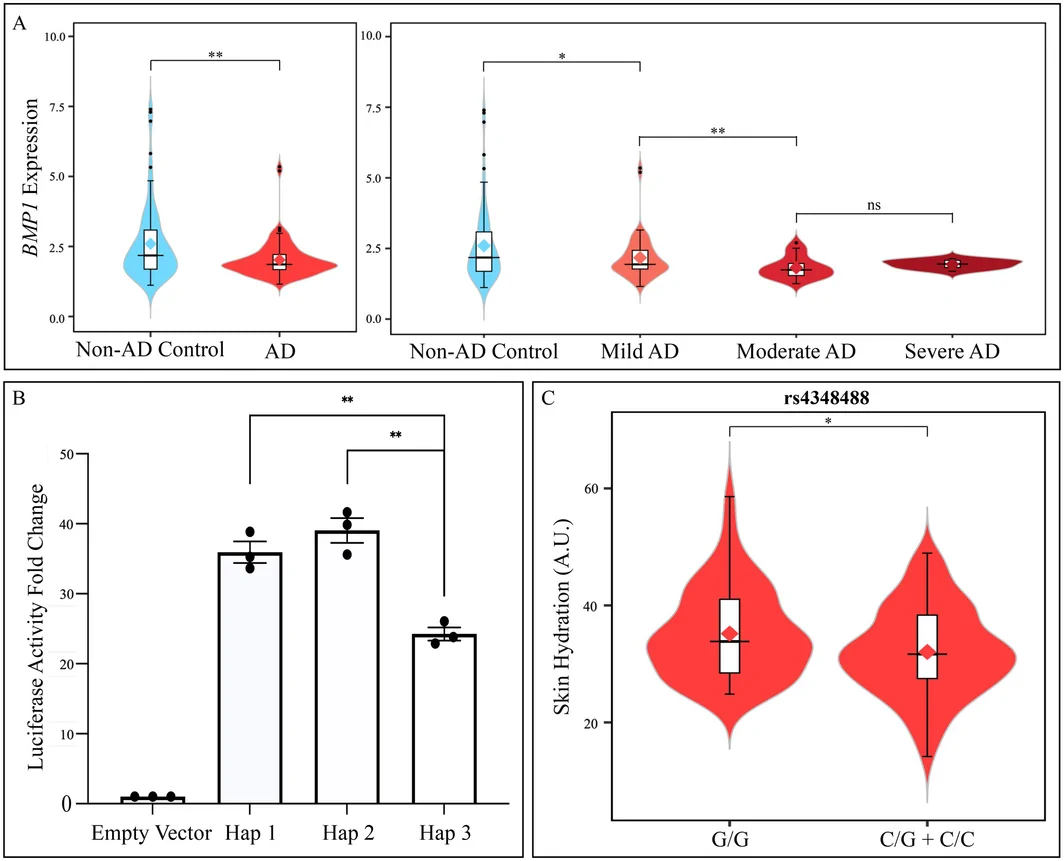
Functional variants at 8p21.3 mediate *BMP1* downregulation, which is associated with lower skin hydration and higher AD risk. (A) *BMP1* transcript expression in peripheral blood mononuclear cells (PBMCs) of AD individuals when compared to non‐AD controls in the Singapore/Malaysia Cross‐Sectional Genetic Epidemiology Study (SMCGES) cohort (*n* = 518). (B) Reporter assay showing fold changes in luciferase activity in human dermal fibroblasts transfected with constructs containing haplotypes 1, 2 or 3 cloned into the pGL4.10 vector. Firefly luciferase activity was normalised to Renilla luciferase activity, and fold change was calculated relative to the empty pGL4.10 vector control. Data represent three independent experiments performed in triplicate and are presented as mean ± standard error of the mean (SEM). (C) Skin hydration (corneometry) readings among AD individuals (*n* = 171), stratified by carrier status of the rs4348488 minor allele ‘C’. A.U., arbitrary units. All statistical significance was assessed using a two‐tailed unpaired *t*‐test unless otherwise mentioned. ns, *p* > 0.05; *, *p* < 0.05; **, *p* < 0.01.

The regulatory mechanism underlying the rs4348488 association with *BMP1* expression was then investigated. Examining variants in LD with rs4348488 implicated rs111596355, located upstream of *BMP1* (Repository Figure S2A) and predicted to alter a transcription factor binding motif. To functionally assess its effect on promoter activity, three haplotypes were constructed, incorporating variants within the 2‐kb region upstream of *BMP1* including rs111596355. They were cloned into pGL4.10 reporter plasmids and transfected into human dermal fibroblasts for luciferase assays. The risk haplotype carrying the rs111596355 minor allele ‘A’ demonstrated significantly reduced promoter activity compared with haplotypes carrying the major allele ‘G’ (*p* < 0.01; Figure 1B), consistent with its association with increased AD risk relative to major‐allele haplotypes (OR = 1.06; 95% CI: 1.02–1.10; *p* < 0.01; Repository Figure S2A). These results indicate that upstream regulatory variants including rs111596355 can directly reduce *BMP1* promoter activity in dermal fibroblasts, explaining the lower *BMP1* expression linked to increased AD susceptibility.

To extend these findings to disease tissue, *BMP1* transcript expression was analysed in AD lesions using the publicly available Gene Expression Omnibus (GEO) dataset GSE120721 [8]. *BMP1* expression was significantly lower in AD patients (*n* = 5) than in healthy controls (*n* = 6; *p* < 0.05; Repository Figure S2B). To assess the relationship between *BMP1* and skin barrier function, skin hydration—a functional readout of barrier integrity and water retention capacity [9]—was measured by corneometry in 171 individuals from the SMCGES genotyped cohort at the antecubital fossa, a site commonly affected in the disease. Among AD individuals, carriers of the rs4348488 minor allele ‘C'—previously associated with lower *BMP1* expression—showed significantly lower corneometer readings than carriers of the major ‘G’ allele (*p* < 0.05; Figure 1C), indicating poorer skin hydration. No genotype‐associated difference was observed among controls (data not shown).

While our findings suggest that regulatory variants at 8p21.3 contribute to AD susceptibility via reduced *BMP1* expression which is associated with lower skin hydration, we acknowledge that our data do not yet establish *BMP1* as a definitive driver of AD. Future genetic perturbation studies in skin‐relevant models with histological and immunostaining analyses will be required to delineate the downstream pathways through which reduced BMP1 activity influences barrier integrity. BMP1 protein expression and localisation should also be validated in AD skin compared with healthy controls.

## Author Contributions

F.T.C. conceived and supervised the study. J.Y.K.W. performed the experiments, analysed the data and wrote the manuscript. Y.Y.S. and Y.Y.E.L. provided advice and guidance on study design and interpretation of results. J.Y.K.W., Y.Y.S., Y.Y.E.L., Y.‐H.S. and K.R. assisted in recruiting study participants and data collation. All authors reviewed and approved the manuscript.

## Funding

Fook Tim Chew (F.T.C.) received grants from the National University of Singapore (N‐154‐000‐038‐001 (E‐154‐00‐0017‐01); C141‐000‐077‐001 (E‐141‐00‐0096‐01)), Singapore Ministry of Education Academic Research Fund (R‐154‐000‐191‐112; R‐154‐000‐404‐112; R‐154‐000‐553‐112; R‐154‐000‐565‐112; R‐154‐000‐630‐112; R‐154‐000‐A08‐592; R‐154‐000‐A27‐597; R‐154‐000‐A91‐592; R‐154‐000‐A95‐592; R‐154‐000‐B99‐114), Biomedical Research Council (BMRC) (Singapore) (BMRC/01/1/21/18/077; BMRC/04/1/21/19/315; BMRC/APG2013/108), Singapore Immunology Network (SIgN‐06‐006; SIgN‐08‐020), National Medical Research Council (NMRC) (Singapore) (NMRC/1150/2008; OFIRG20nov‐0033; MOH‐001636 (OFLCG23may‐0038, A‐8002641‐00‐00)), National Research Foundation (NRF) (Singapore) (NRF‐MP‐2020‐0004), Singapore Food Agency (SFA) (SFS_RND_SUFP_001_04; W22W3D0006; NRF‐SFSRND2SIH‐0001; SFS_RND_2_FS_0002), Singapore's Economic Development Board (EDB) (A‐8002576‐00‐00), and the Agency for Science Technology and Research (A*STAR) (Singapore) (H17/01/a0/008; and APG2013/108). This research is supported by the National Research Foundation Singapore under its Open Fund‐Large Collaborative Grant (MOH‐001636) (A‐8002641‐00‐00) and administered by the Singapore Ministry of Health's National Medical Research Council. Kavita Reginald (K.R.) has received funding from the T20 Research Collaboration Grant Scheme from Sunway University with Grant No.: STR‐RMF‐T20‐005‐2019. The funding agencies had no role in the study design, data collection and analysis, decision to publish or preparation of the manuscript.

## Ethics Statement

The study conducted across both Singapore and Malaysia was carried out in accordance with the Declaration of Helsinki. Ethical approval was obtained from the Institutional Review Board of the National University of Singapore (NUS; 07‐023, 09‐256, 10‐445, 13‐075, 14‐150, 18‐036), the Scientific and Ethical Review Committee of the Universiti Tunku Abdul Rahman (UTAR; U/SERC/03/2016) and the Sunway University Research Ethics Committee (SUREC 2019/029). Written informed consent was obtained from all participants. For those under 21 years of age, additional consent was obtained from a parent or legal guardian.

## Conflicts of Interest

F.T.C. reports grants from the National University of Singapore, Singapore Ministry of Education Academic Research Fund, Singapore Immunology Network, National Medical Research Council (NMRC) (Singapore), Biomedical Research Council (BMRC) (Singapore), National Research Foundation (NRF) (Singapore), Singapore Food Agency (SFA), Singapore's Economic Development Board (EDB) and the Agency for Science Technology and Research (A*STAR) (Singapore), during the conduct of the study; and consulting fees from Sime Darby Technology Centre; First Resources Ltd.; Genting Plantation, Olam International, Musim Mas and Syngenta Crop Protection outside the submitted work. The other authors declare no other competing interests.

## Data Availability

The data supporting the findings of this study are available at https://osf.io/f2zvk/overview?view_only=efca34ae7a9c457dbefa01ff4e8ef9da. All other data used in this study are available from the corresponding author (F.T.C.) upon resonable request.
